# Clinical features of hepatocellular carcinoma with hepatitis B virus among patients on Nucleos(t) ide analog therapy

**DOI:** 10.1186/s13027-020-0277-y

**Published:** 2020-02-04

**Authors:** Li Liu

**Affiliations:** Infectious disease department, Hunan provincial peoples ~ hospital (The first affiliated hospital of Hunan Normal university), Hunan province, Changsha, 410000 China

**Keywords:** Hepatocellular carcinoma, HBV, Nucleos(t) ide analogs

## Abstract

**Background:**

The clinical manifestation of hepatocellular carcinoma (HCC) with hepatitis B virus (HBV) varies significantly between patients treated with or without nucleos(t) ide analog (NUC) therapy. To have a better understanding of HCC with HBV, we compared the clinical features of patients with HCC receiving or not receiving NUC therapy.

**Methods:**

We retrospectively reviewed the medical records of 76 patients with HBV-caused HCC who received treatment at the Hunan Provincial Peoples’ Hospital starting from January 1, 2008 to December 31, 2017. They were categorized into two groups, namely, NUC group and non-NUC group, based on whether they had received NUC therapy or not.

**Results:**

The percentage of liver pain (36.36% vs. 79.07%; *p* < 0.05) and appetite loss (30.30% vs. 70.27%; *p* < 0.05) in the NUC group was lower than that in the non-NUC group. We observed a similar trend for the percentage of undetectable HBV-DNA (11.63% vs. 63.64%; *p* < 0.05) and normal ALT (25.58% vs. 75.76%; *p* < 0.05) between non-NUC and NUC groups. There were no significant differences between the two groups with respect to TBiL (*p* = 0.370) and ALB (*p* = 0.475). The same trend was observed for the proportion of HBeAg negative (*p* = 0.719) and AFP ≤ 14.65 ng/mL (*p* = 0.199) in both groups. Single tumor nodule was more common in the NUC group compared to the non-NUC group (66.67% vs. 6.98%; *p* < 0.05). An opposite trend was observed for portal vein invasion (18.18% vs. 79.07%; *p* < 0.05) and metastasis (0% vs. 44.19%; *p* < 0.05).

**Conclusions:**

Nucleos(t) ide analog therapy exerts a significant impact on the clinical and radiological characteristics of hepatocellular carcinoma with HBV. Patients receiving nucleos(t) ide analog therapy were found to have milder symptoms and fewer radiographic findings.

## Background

Hepatocellular carcinoma (HCC) has been reported to be the most frequent primary liver malignancy and the third cause of cancer-related deaths worldwide [1]. Despite the fact that HCC does not top the list of most frequent cancer types, its high mortality is associated with its poor resectability, high chance of relapse after surgical resection, and poor response to conventional treatment, making it a global health problem [2]. It has a higher incidence in males than in females (~ 2.4:1). Geographically, it occurs more frequently in individuals in Eastern and Southern Asia, and Middle and Western Africa [3]. Without exception, HCC accounts to be a great threat to peoples’ health in China as well [4, 5].

Up to 70 to 90% of patients with HCC have an established history of chronic liver disease and liver cirrhosis. Dominant risk factors for the development of cirrhosis include chronic infection with Hepatitis B Virus (HBV), Hepatitis C Virus (HCV), Hepatitis delta virus (HDV), non-alcoholic steatohepatitis (NASH), high intake of alcohol and exposure to aflatoxin B1, diabetes, obesity [2, 6–9]. Among these factors, chronic infection with HDV has been reported to correlate with a deterioration of HBV infection, resulting in a higher rate of developing cirrhosis, liver decompensation, and HCC [6]. Globally, HBV accounts for approximately 80% of virus-correlated HCC cases, which are mainly confined to Africa and East Asia, making these the topmost HCC incidence regions. However, HCV-caused HCC accounts for approximately 20% of virus-associated HCC cases, which are mainly reported in Western Europe and North America [2, 10]. Chronic HBV infection is one of the leading risk factors for HCC worldwide, accounting for at least 50% cases of primary liver tumors. Although HBV is the main causative agent in the high incidence HCC areas [2], it has been reported that the risk of developing HCC is 15 to 20 times greater among HBV-infected individuals as compared to the uninfected population [11, 12].

The development of nucleos(t) ide analogs (NUC) has influenced the management of hepatitis B virus infection. Treatment of chronic hepatitis B (CHB) infection with oral antiviral drugs, such as tenofovir and entecavir, is recommended in patients with cirrhosis and those with severe hepatitis and/or fibrosis [13]. Long-term monotherapy with one of the current first-line nucleos(t) ide analogs (NAs), entecavir (ETV) or tenofovir disoproxil fumarate (TDF), results in maintained virologic remission in more than 95% of patients, often achieving regression of histologic lesions of cirrhosis and preventing or reversing hepatic decompensation [14]. NUC has been shown to decrease the risk of developing HCC [15, 16]; however, patients with chronic HBV are still at the risk for developing HCC [17, 18], even when they have maintained virologic remission under long-term therapy with NA(s), particularly in patients with cirrhosis [19, 20]. The annual incidence rate during NUC therapy has been reported to be 0.3 to 1.2% in non-cirrhosis cases and 1.8 to 6.0% in cirrhosis cases, indicating that the suppressive effect of NUC therapy on HCC would be insufficient [21]. There are few reports about clinical features of HCC with HBV among patients with NUC therapy. In the present study, we analyzed the clinical features, imaging, and prognosis of HCC patients with HBV who had undergone NUC therapy.

## Methods

The present study was a retrospective study conducted at the Hunan Provincial People’s Hospital. Records of HBV patients with definite HCC who were admitted during a 10-year period (from January 1, 2008, to December 31, 2017) were examined. Patients’ demographics, clinical manifestations, computed tomographic (CT) data, diagnosis, treatment, and prognosis were analyzed. The relevant follow-up data were obtained through regular clinical interviews or via telephone calls. The last follow-up information was collected on December 15, 2018.

### Patients

All patients who were diagnosed as HCC with HBV from January 1, 2008, to December 31, 2017, at our inpatient department at the Hunan Provincial People’s Hospital were enrolled in this study. On the basis of whether they had received NUC therapy or not, they were divided into two groups, namely, NUC group and non-NUC group. The NUC group consisted of HCC patients diagnosed in the first half-year after the onset of NUC therapy. In both groups, patients who showed co-infection with hepatitis D, hepatitis C or human immunodeficiency virus, and those who had undergone liver transplantation were excluded. Moreover, other co-factors of liver disease, such as alcohol intake and diabetes, were also excluded.

In the NUC group, all patients received anti-HBV treatment under NUC therapy: 14 patients with entecavir (ETV), nine patients with lamivudine, seven patients with adefovir, two patients with a combination of lamivudine and adefovir, and one patient with a combination of ETV and adefovir. Two patients had received interferon treatment for one year; ten years and eight years ago, respectively. The NUC therapy was initiated 57 (range: 6–120) months before the development of HCC. The initial NUC therapy was lamivudine, ETV, or adefovir as monotherapy. For patients resistant to lamivudine, adefovir 10 mg/d was added since 2009. For those resistant to adefovir, ETV 0.5 g/d was added or transferred since 2010.

The following clinical data obtained at presentation of HCC were collected: age (year), gender, antiviral agent, serum albumin or bilirubin, serum alanine aminotransferase (ALT, IU/L) levels, serum alpha-fetoprotein (AFP) levels, presence of liver cirrhosis, and HBe antigen (HBeAg), HBV DNA (Copies/mL) levels.

### Diagnosis

A definite diagnosis of HCC was made if the HBV patients met the following conditions: for nodules > 1 cm in cirrhosis or chronic hepatitis B, characteristic vascular patterns on a 4-phase CT, HCC could be diagnosed without biopsy (the characteristic vascular patterns are defined as arterial hypervascular and venous or delayed phase washout) [22].

### Statistical analysis

The chi-square test was used for inter-group comparisons with categorical variables. Continuous variables were analyzed by the independent sample’s *t*-test. All data were analyzed with SPSS Version 20.0. A *p-*value < 0.05 was considered to be statistically significant.

## Results

### Demographic characteristics of the patients

We examined the records of 76 patients who were diagnosed with HCC with HBV from January 1, 2008, to December 31, 2017. Among these, 33 patients had received NUC therapy; the others had not received the NUC therapy. There were no significant differences between the two groups with respect to gender (96.97% vs. 95.45%; *p* = 0.849) and age (53.06 ± 2.14 vs. 52.87 ± 2.08; *p* = 0.952).

### Clinical features

At the presentation of HCC, 12 patients reported pain in the liver area in the NUC group, whereas 34 patients in the non-NUC group reported pain (36.36% vs. 79.07%; *p* < 0.05). Ten patients in the NUC group reported appetite loss, whereas 30 patients reported the same in the non-NUC group (30.30% vs. 70.27%; *p* < 0.05). There was no difference between the two groups in the incidence of clinical symptoms, such as fatigue (39.39% vs. 51.16%; *p* = 0.308), vomiting (6.06% vs. 4.65%; *p* = 0.785). With respect to TBil (31.39 ± 5.96 vs. 43.37 ± 11.37; *p* = 0.370) and ALB (35.76 ± 1.26 vs. 35.39 ± 0.94; *p* = 0.475), there were no differences between the two groups. Six patients in the NUC group and 21 patients in the non-NUC group had undetectable HBV DNA (11.63% vs. 63.64%; *p* < 0.05). Thirty-two patients were HBeAg negative in the NUC group, whereas 41 patients were HBeAg negative in the non-NUC group (96.97% vs. 95.35%; *p* < 0.05). Twenty-five patients had normal ALT levels in the NUC group, and 11 patients had normal ALT levels in the non-NUC group (75.76% vs. 25.58%; *p* < 0.05). The AFP levels were ≤ 14.65 ng/mL in 39.39% of patients in the NUC group, whereas there were 25.58% patients with AFP ≤ 14.65 ng/mL in the non-NUC group (39.39% vs. 25.58%; *p* = 0.199). There were no differences between the two groups (Table 1).

**Table 1:** Clinical and virologic characteristics of HCC patients in NUC group and non-NUCl group

	NUC group *n* = 33(%)	Non-NUC group *n* = 43(%)	*P* value
Liver area pain	12(36.36%)	34(79.07%)	< 0.05
Fatigue	13(39.39%)	22(51.16%)	0.308
Vomit	2(6.06%)	2(4.65%)	0.785
Appetite loss	10(30.30%)	30(70.27%)	< 0.05
TBIL (umol/L)	31.39 ± 5.96	43.37 ± 11.37	0.370
ALB(g/L)	35.76 ± 1.26	35.39 ± 0.94	0.475
HBeAg negative	32(96.97%)	41(95.35%)	0.719
HBV-DNA undetectable	21(63.64%)	6(11.63%)	< 0.05
ALT< 40 U/L	25(75.76%)	11(25.58%)	< 0.05
AFP (ng/ml) < 14.65	13(39.39%)	11(25.58%)	0.199

### Computed tomography

The computed tomography (CT) scan data were collected from 76 patients. The characteristics of the images are given in Table 2. Twenty-two patients had a single tumor node in the NUC group, whereas three patients had a single tumor node in the non-NUC group (66.67% vs. 6.98%, *p* < 0.05). Twenty-three patients were included in the Milan criteria in the NUC group, and five patients were included in the Milan criteria in the non-NUC group (69.70% vs. 11.63%; *p* < 0.05). There were fewer tumor portal vein invasions and no metastasis in the NUC group; most patients had portal vein invasion and tumor metastasis in non-NUC group. Moreover, there were statistically significant differences between the NUC and non-NUC groups when we compared portal vein invasion (18.18% vs. 79.07%; *p* < 0.0) and metastasis (0% vs. 44.19%; *p* < 0.05).

**Table 2:** Characteristics of tumors and radiological patterns of abnormalities CT scan of HCC patients in NUC group and non-NUC group

	NUC group *n* = 33(%)	Non-NUC group *n* = 43(%)	*P* value
Solitary nodule	22(66.67%)	3(6.98%)	< 0.05
Multiple nodules	11(33.33%)	40(93.02%)	< 0.05
Portal vein invasion	6(18.18%)	34(79.07%)	< 0.05
Metastasis	0(0)	19(44.19%)	< 0.05
In Milan criteria	23(69.70%)	5(11.63%)	< 0.05

### Treatment

The treatment included liver transplantation, liver resection, transarterial chemoembolization (TACE), micro-ablation, radiation therapy, and administration of sorafenib. The effective therapy was any potentially curative treatment in 27 patients in the NUC group: two patients received liver transplantation; two patients received liver resection monotherapy; 17 patients received liver TACE multiple times (from 1 to 8 times); two patients received liver resection and TACE; two patients received micro-ablation monotherapy; two patients received micro-ablation and TACE; and six patients quit the undergoing treatment owing to economical reasons. Six patients received effective treatment in the non-NUC group: one patient received liver resection monotherapy; two patients received liver resection and TACE; two patients received TACE for multiple times (from 1 to 5 times); and one patient received sorafenib. Thirty-seven patients did not receive any effective treatment because of metastasis and poor body status (Table 3).

**Table 3:** Treatment of HCC patients in NUC group and non-NUC group

	NUC group *n* = 33(%)	Non-NUC group *n* = 43(%)
Liver transplantation	2(6.06%)	0(0)
Liver resection	2(6.06%)	1(2.33%)
Liver resection+TACE	2(6.06%)	2(4.65%)
TACE	17(51.52%)	2(4.65%)
Ablation	2(6.06%)	0(0)
TACE add ablation	2(6.06%)	0(0)
Sorafenib	0(0)	1(2.32%)
No treatment	6(18.18%)	37(86.04%)

### Prognosis

The outcome was 90-day all-cause mortality; mortality attributable to liver cancer was also determined. In the NUC group, 3 (9.09%) patients died in 90 days; 30 (90.91%) patients survived for more than 90 days, 15 (45.45%) patients survived for more than 1 year; and 10 (30.30%) patients survived for more than two years. In the non-NUC group, 29 (67.46%) patients died in 90 days, most of them had serious dyscrasia during follow-up, and 14 (32.54%) patients survived for more than 90 days (Table 4). During 60 (range: 0–120) months after the diagnosis of HCC in the NUC group, 26 (78.79%) patients died; all of them died due to HCC progression: 17/26 BCLC 0/A, 3/26 BCLC B and 6/26 BCLC C. No HCC patient died of other reasons such as cardio-cerebral vascular problems, tumors with other organ origins or traffic accidents.

**Table 4:** Prognosis of 76 patients with HCC

	Anti-viral group *n* = 33(%)	Non anti-viral group *n* = 43(%)	*P* value
Prognosis			
Survival	30(90.91%)	14(32.54%)	< 0.05
Death	3(9.09%)	29(67.46%)	

## Discussion

Recently, nucleos(t) ide analogs have started playing important roles in protecting against liver injury, prolonging the deterioration of liver cirrhosis, and reducing the occurrence of HCC in patients with HBV [23]. Nonetheless, as the duration of antiviral treatment increases, the risk for HCC remains despite undetectable HBV-DNA in the serum [24]. This is because it is unable to act on integrated viral sequences and covalently closed circular HBV DNA, both factors contributing to chromosomal instability and activation of cancer-related genes and inactivation of protective genes. In the study by Yoo J [25], some patients had negative serum HBV-DNA for over 12 years before developing HCC. In our study, 21 patients developed HCC with negative serum HBV-DNA for several years in the NUC group, the results being similar to those of Yoo’s study.

The present study provides several new findings. At first, nearly 70% HCC patients in the NUC group were asymptomatic, and their disease was detected incidentally during the follow-up. The most common symptom of HCC was liver area pain. The proportion of liver area pain in patients with NUC therapy (36.36%) was lower than that in patients with non-NUC therapy (79.07%). Second, the negative diagnostic accuracy of AFP levels was the same between the two groups; there was no difference. AFP levels were normal in almost one-third of patients. There was no relationship with NUC therapy. In contrast to this finding, data from a previously published study from Korea reported that patients with current or recent exposure to NUC therapy showed poorer performance of AFP, when compared with untreated patients [26]. Finally, the most common CT findings of HCC in the current study were the presence of nodules, portal vein invasion, and metastasis, the radiological patterns of HCC occurring in the NUC group compared favorably with patients in the non-NUC group. The result showed that solitary nodule was significantly more common in patients in the NUC group. Loglio reported that single, small tumors were more common in patients under long-term NUC treatment [27], a finding similar to that of the current study. Multiple nodules were significantly more common in the non-NUC group, because long-term effective anti-HBV oral therapy can reduce the inflammation and fibrosis of the liver, and the effective control of HBV DNA replication reduces the release of cytokines and growth factors, thus reducing the necrosis of hepatocytes and proliferation of fibroblasts and lowering the high turnover of hepatocytes. Inactive hepatitis reduces the risk of host DNA mutations, responsible for their malignant transformation [28]. The portal vein invasion was significantly less frequent in patients with NUC therapy. Wei found that HCC with portal vein tumor thrombus invading the main trunk showed a significantly less rate of receiving antiviral treatment, which is similar to our finding. Other common findings, such as metastasis, were significantly more frequent in patients with non-NUC therapy.

Liver resection remains the main curative option for HCC [29]. However, TACE is the most widely utilized and is considered the first-line treatment for patients staged as intermediate HCC (Barcelona Clinic Liver Cancer stage B) [30]. In the NUC group, 54.55% of patients received TACE and 12.12% of patients received TACE and ablation; 45.45% of patients survived for more than 1 year, 51.52% of patients received TACE ,6.06% of patients received ablation;and 6.06% of patients received TACE add ablation. It showed that TACE, ablation and TACE ablation were effective treatments for HCC. Moreover, the study by Hu reported that MWA-TACE (microwave ablation-transarterial chemoembolization) was a safe, feasible, and effective therapy for the treatment of 5.0-cm to 10.0-cm HCC lesions in patients with cirrhosis [31]. Similarly, Wang found that TACE combined with partial hepatic segment thermal ablation was a safe and effective treatment for liver cancer patients, especially for those with more advanced disease [32].

Mortality was markedly lower in patients with NUC therapy (9.09%) than in patients with non-NUC therapy (67.46%) in 90 days. There was an increased relationship with surveillance. As we know, patients with NUC therapy should be examined regularly, usually at an interval of 3 to 6 months; this may make access to surveillance easier. Seventy percent of patients within the “Milan criteria” in the NUC group were assured the best treatment options (effective treatment options offered to 82% patients) and the best prognosis.

The present study had certain limitations. First, it was a retrospective study and examined a small number of patients with HCC. Second, the above-mentioned NUC therapy with HBV indicated only one aspect of the disease and lacked a dynamic evolution process.

## Conclusions

The results of the present study demonstrated that the NUC therapy had a significant impact on the clinical and radiological characteristics of HCC with HBV. Patients with HCC on NUC therapy were found to have lesser symptoms and fewer radiographic findings than those not undergoing the therapy.

## Data Availability

All data generated or analyzed during this study are included in the published article.

## Abbreviations

CT: Computed tomography
HBV: Hepatitis B virus
HCC: Hepatocellular carcinoma
MWA –TACE: Microwave ablation-transarterial chemoembolization
NUC: Nucleos(t) ide analogs
TACE: Transarterial chemoembolization
